# Hämatoonkologie und Intensivmedizin: Vom Tabu zur Conditio sine qua non

**DOI:** 10.1007/s00063-020-00737-5

**Published:** 2020-10-12

**Authors:** P. Wohlfarth, P. Schellongowski, Arbeitsgruppe für Hämato-Onkologische Intensivmedizin der Österreichischen Gesellschaft für Allgemeine und Internistische Intensivmedizin und Notfallmedizin (ÖGIAIN); Initiative „Intensive Care in Hematologic and Oncologic Patients“ (iCHOP)

**Affiliations:** Intensivstation 13i2, Universitätsklinik für Innere Medizin I, Medizinische Universität Wien, Währinger Gürtel 18–20, 1090 Wien, Österreich

**Keywords:** Krebs, Intensivstation, Akutes respiratorisches Versagen, Immuntherapie, iCHOP, Cancer, Intensive care unit, Acute respiratory failure, Immunotherapy, iCHOP

## Abstract

Intensivmediziner werden im Kontext der Versorgung von kritisch kranken Krebspatienten vor eine zunehmende Bandbreite spezifischer Herausforderungen gestellt. Neben einer adäquaten Therapiezielfindung umfasst diese die Versorgung des akuten respiratorischen Versagens (ARV) mit speziellen differenzialdiagnostischen Überlegungen, das Management immunologischer Nebenwirkungen innovativer Krebstherapien sowie eine Vielzahl an Krankheitsbildern, die ausschließlich bei Krebspatienten auftreten. Um diesen Herausforderungen gerecht werden zu können, widmet sich die Initiative „Intensive Care in Hematologic and Oncologic Patients (iCHOP)“ seit einigen Jahren diesen Themen. Unterstützt durch mehrere österreichische und deutsche Fachgesellschaften für Intensivmedizin, Hämatologie und Onkologie wurde kürzlich der „1. Konsens zur Versorgung kritisch kranker Krebspatienten“ mit Empfehlungen zum klinischen Management sowie infrastrukturellen und ausbildungsassoziierten Themen verfasst. Das Auftreten eines ARV steht bei kritisch kranken Krebspatienten seit jeher im Fokus der Forschung. Während die nichtinvasive Beatmung lange als Goldstandard der Therapie galt, zeigen hochqualitative Studien jedoch keine relevanten klinischen Vorteile dieser Techniken inklusive der High-flow-nasal-oxygen-Therapie im Vergleich zur konventionellen Sauerstofftherapie. Hingegen rückt eine nichtgeklärte Ätiologie des ARV als *einziger* potenziell modifizierbarer Risikofaktor in den Fokus. Dementsprechend sind evidenzbasierte und rigoros angewendete Diagnosealgorithmen bei diesen Patienten von eminenter Bedeutung. Des Weiteren stellen das Erkennen und das Management der immer häufiger vorkommenden vielgestaltigen immuntherapieassoziierten Toxizität Intensivmediziner vor zunehmende Herausforderungen.

## Abstract (en)

Intensivists are confronted with a broad spectrum of specific clinical problems while caring for critically ill cancer patients. These include defining proper goals of intensive care treatment, managing acute respiratory failure with diverse differential considerations, treating immunologic side-effects of ever new and innovative cancer therapies, as well as numerous clinical scenarios which may exclusively arise in cancer patients. To help clinicians handle such challenges, the initiative Intensive Care in Hematologic and Oncologic Patients (iCHOP) has been dealing with these topics for several years. Supported by several Austrian and German medical societies of intensive care medicine, hematology and oncology, the first “Consensus statement for cancer patients requiring intensive care support” has only recently been released. Acute respiratory failure and its management continues to be a major focus in critically ill cancer patients due to its frequency and its prognostic impact. While noninvasive oxygenation strategies were considered the gold standard of therapy, more recent high-quality data do not show clinical benefits of such techniques including high flow nasal oxygen. On the contrary, several studies revealed an unidentified etiology of an acute respiratory failure as the *only* potentially modifiable risk factor for adverse outcome. Consequently, evidence-based and rigorously applied diagnostic algorithms are of utmost importance in these patients. Furthermore, intensivists are increasingly confronted with the rising incidence of various and new immunotherapy-associated toxicities and their management.

Die Versorgung kritisch kranker Krebspatienten erfordert durch die große Bandbreite spezifischer Problemstellungen mit ständiger Dynamik eine zunehmende Spezialisierung von intensivmedizinisch tätigen Ärzten (Tab. 1). Der erste Konsensus deutschsprachiger intensivmedizinischer und hämatologisch-onkologischer Fachgesellschaften spricht auf dem Boden der vorhandenen Evidenz Empfehlungen zum klinischen Management aus [1]. Dieser Review behandelt neben allgemeinen Themen speziell rezente Daten zur Oxygenierungsstrategie bei akutem respiratorischem Versagen (ARV), die Wichtigkeit einer rigorosen Ursachenabklärung sowie die zunehmend auftretenden Toxizitäten nach Immuntherapie.

**Table 1:** 

Akutes (hypoxisches) respiratorisches Versagen
Infektiologische Probleme und Notfälle
Arzneimittelreaktionen nach Immun- oder Chemotherapie
Tumorlysesyndrom
Hyperleukozytosesyndrom
Hyperviskositätssyndrom
Elektrolytstörungen (SIADH, Hyperkalzämie)
Komplikationen nach Stammzell- und Knochenmarktransplantation
Thrombotische Mikroangiopathien
Thrombophilie/hämorrhagische Diathese
Hämophagozytosesyndrom
Maligne Atemwegsobstruktion
Oberes Vena-cava-Syndrom
Malignes spinales Kompressionssyndrom
Zerebrale Metastasen

*ICU* „intensive care unit“; *SIADH* „syndrome of inappropriate antidiuretic hormone secretion“

## Therapiezielfindung

Die vorhandene Literatur zeigt konklusiv, dass sich das Outcome kritisch kranker Krebspatienten in den vergangenen Jahrzehnten stetig verbessert hat [2, 3]. Diese Tatsache ist

- 1) auf ein verbessertes intensivmedizinisches Management analog zu nichtimmunsupprimierten Patienten,
- 2) auf spezifische Fortschritte und Erkenntnisse bei kritisch kranken Krebspatienten, beispielsweise im Infektmanagement und in der Verabreichung von systemischen Therapien bei malignombedingten akuten Organversagen sowie
- 3) maßgeblich auf eine verbesserte Therapiezielfindung in dieser Patientengruppe

zurückzuführen [1, 4, 5]. So bedeutet eine „inadäquate“ Entscheidung im Rahmen einer Intensivstationsaufnahme im Fall einer Überbehandlung möglicherweise eine für den Patienten und seine Familie sinnlos belastende Erfahrung am Lebensende. Hingegen kann eine Ablehnung bei bestehenden onkologischen Therapieoptionen hinsichtlich des Malignoms eine fatale vertane Chance darstellen.

### „Full-code“-Management

An prinzipiellen Therapiezieloptionen besteht das „Full-code“-Management ohne Zurückhaltung intensivmedizinisch vorhandener Therapieoptionen. Diese Kategorie trifft in der Regel auf Patienten mit kurativen Therapieansätzen, Patienten in Remission, sowie Patienten mit beträchtlicher Langzeitüberlebenswahrscheinlichkeit, also eventuell auch im aus onkologischer Sicht nichtkurativen, palliativen Setting in Abhängigkeit etwaiger Komorbiditäten zu [1, 4].

### „No-ICU“-Entscheidung

Im konträren Gegensatz dazu sind „No-ICU“-Entscheidungen in der Regel die zutreffendste Option für Patienten, die einen sehr schlechten Performancestatus haben (mit Ausnahme von Situationen, in denen spezifische Therapien Aussicht auf substanzielle Besserung bieten), für die keine lebensverlängernden Therapien bestehen, sowie für Patienten, die eine intensivmedizinische Behandlung ablehnen [1, 4].

### ICU Trial

Für Patienten mit „intermediärer Prognose“ kann ein ICU Trial, also ein zeitlimitiertes „Full-code-Management“, das angemessene Herangehen darstellen, wobei die adäquate Dauer solcher „Trials“ schwer zu bestimmen ist, in der Regel jedoch mehrere Tage nicht unterschreiten sollte [6].

Die vertrauensvolle Zusammenarbeit zwischen Intensivmedizinern und Krebsspezialisten, insbesondere im Kontext weniger klarer Situationen hinsichtlich der malignomassoziierten Prognose, ist eine entscheidende Voraussetzung für diesen Therapiezielfindungsprozess [7].

### Timing der Intensivstationsaufnahme

Die vorhandenen Daten über kritisch kranke Krebspatienten mit den häufigsten Intensivstationsaufnahmegründen, ARV und Sepsis, zeigen einmütig, dass eine möglichst frühzeitige Verlegung auf die Intensivstation, also bei manifestem oder eventuell auch nur drohendem Organversagen, erfolgen sollte [8, 9]. Um einen solchen frühen Zeitpunkt nicht zu übersehen, empfiehlt es sich, bereits im Umfeld der Normalbettenstation ein regelmäßiges Screening bezüglich Sepsiskriterien und des Auftretens von Organversagen durchzuführen [1].

## Akutes respiratorisches Versagen

### Beatmung

Die Beatmung eines onkologischen Patienten mit ARV stellt einen Paradigmenwechsel dar. Die zur Verfügung stehenden Optionen werden nachfolgend dargestellt.

#### Nichtinvasive Beatmung

Bei Patienten mit „chronic obstructive pulmonary disease“ (COPD) und hypertensivem Lungenödem stellt die nichtinvasive Beatmung (NIV) den Goldstandard der Therapie dar und es gibt keine evidenzbasierte Rationale, dass dies nicht auch bei Krebspatienten gelten sollte. Bei Krebspatienten mit *akutem hypoxischen* respiratorischen Versagen (ARV) ist die Rolle der NIV jedoch zunehmend weniger gut belegt. Eine ältere randomisierte Studie zeigte zwar die Überlegenheit einer NIV gegenüber einer konventionellen Sauerstofftherapie bei Immunsupprimierten (zumeist hämatologischen Patienten) hinsichtlich Intubations- und Mortalitätsraten [10]. Im Einklang damit zeigte eine Metaanalyse mit insgesamt 2380 immunsupprimierten, in erster Linie hämatologischen Patienten, dass eine initiale NIV im Vergleich zur Intubation mit niedrigerer Mortalität vergesellschaftet ist. Jedoch mussten 61 % (Interquartilsabstand [IQR] 40–78 %), also nahezu 2 von 3 Patienten, nach initialer NIV sekundär intubiert werden, was mit einer *erhöhten* Mortalität assoziiert war [11]. Im Rahmen einer weiteren multizentrischen Beobachtungsstudie trat bei 1004 Immunsupprimierten mit ARV, erneut überwiegend Krebspatienten, in 71 % der Fälle NIV-Versagen auf, was ebenfalls mit erhöhter Mortalität vergesellschaftet war [12].

Die nichtinvasive Beatmung zeigte in einer multizentrischen Studie weder Vorteile noch Nachteile

Eine große multizentrisch randomisiert-kontrollierte Studie, in der NIV mit konventioneller Sauerstofftherapie bei Immunsupprimierten (vor allem hämatologischen) Patienten mit akutem hypoxischen ARV, konnte keine klinischen Vorteile (aber auch keine Nachteile) einer NIV zeigen. Ein akutes kardiales Lungenödem oder eine Hyperkapnie, i.e. p_a_CO_2_ >50 mm Hg, stellten bei dieser Studie Ausschlussgründe dar. Die unerwartet niedrige Mortalität in der O_2_-Gruppe führte jedoch dazu, dass die Studie eine zu geringe Power aufwies. Darüber hinaus erhielten mehr Patienten in der NIV-Gruppe im Vergleich zur O_2_-Gruppe intermittierend nasalen High-flow-Sauerstoff (HFNO), sodass die NIV-Gruppe möglicherweise nicht repräsentabel war [13].

#### Nasale High-flow-Sauerstoffgabe

Während sich in der Analyse der sekundären Outcomevariablen der FLORALI-Studie (HFNO bei hypoxischem ARV, eine prinzipiell negative Studie!) Hinweise ergaben, dass HFNO im Vergleich zur gewöhnlichen O_2_-Gabe oder NIV mit einer niedrigeren Intubations- und Mortalitätsrate bei älteren Patienten mit Pneumonie [14] und in einer Post-hoc- Analyse auch bei immunsupprimierten Patienten [15] vergesellschaftet sein *könnte*, konnte eine aktuelle große Studie mit knapp 800 immunsupprimierten Patienten und hypoxischem ARV keinen Unterschied zwischen HFNO und O_2_-Insufflation hinsichtlich Intubations- und Mortalitätsraten zeigen [16]. Interessanterweise wurden in dieser bis dato größten Studie zum Einsatz der HFNO bei hypoxischem ARV auch keine Unterschiede hinsichtlich der Symptomatik der Patienten nachgewiesen.

Die Mortalität primär intubierter immunsupprimierter ARV-Patienten ist deutlich verbessert

Etliche Studien zeigen eine deutlich verbesserte Mortalität von primär intubierten immunsupprimierten Patienten mit ARV. So lag beispielweise die Mortalität bei ARV mit Kriterien eines „acute respiratory distress syndrome“ (ARDS) bei invasiv beatmeten Patienten bei „nur mehr“ 52 % [12].

Zusammenfassend bestehen bei deutlich gesunkenen Mortalitätsraten invasiv beatmeter Krebspatienten mit ARV in den vergangenen Jahren keine eindeutigen Hinweise mehr für die Vorteile einer NIV oder HFNO im Vergleich zu einer konventionellen O_2_-Insufflation. Das ehemalige Paradigma, immunsupprimierte Patienten nach Möglichkeit und womöglich „um jeden Preis“ nicht zu intubieren, kann somit als überholt, ja im Fall sekundärer Intubationen bei NIV-Versagen sogar als potenziell gefährlich angesehen werden.

Die Intubation und Beatmung soll bei Indikation ohne Verzögerung erfolgen

Konträr dazu kann nunmehr der Grundsatz formuliert werden, dass Krebspatienten mit aus intensivmedizinischer Sicht kurativer Therapiezielsetzung (also keine Therapielimitierungen hinsichtlich des Einsatzes intensivmedizinischer Therapiemodalitäten) bezüglich der Oxygenierungsstrategie nicht anders als Nichtkrebspatienten geführt werden sollten.

Folgerichtig empfiehlt der „Konsensus zur Versorgung kritisch kranker Krebspatienten“ der Initiative „Intensive Care in Hematologic and Oncologic Patients“ (iCHOP), unterstützt von der Österreichischen Gesellschaft für Internistische und allgemeine Intensiv- und Notfallmedizin (ÖGIAIN), der Österreichischen Gesellschaft für Hämatologie und Onkologie (ÖGHO), der Deutschen Gesellschaft für Internistische Intensiv- und Notfallmedizin (DGIIN) sowie der Deutschen Gesellschaft für Hämatologie und Onkologie (DGHO) in freier Übersetzung: *„Falls ein Behandlungsversuch mit NIV oder HFNO bei Krebspatienten mit akutem ARV eingeleitet wird, sollten die wesentlichen Kontraindikationen und/oder das Auftreten präspezifizierter Intubationskriterien beachtet werden und bei Vorliegen dieser Kriterien die Intubation und Beatmung ohne Verzögerung erfolgen (A-IIu)“ *[1]*. *Außerdem wird von einem NIV- oder HFNO-Versuch in einem Nichtintensivstationssetting abgeraten (*„Angesichts der beträchtlichen Versagensraten von NIV und HFNO bei Krebspatienten mit einem hypoxischen ARV und fehlender suffizienter Daten zur Sicherheit dieser Methoden auf Normalstationen sollten NIV und HFNC bei dieser Indikation auf Normalstationen nicht zum Einsatz kommen (B-III).“*; [1])*.*

### Diagnostik und spezifische Therapie

Die Differenzialdiagnosen des ARV bei kritisch kranken Krebspatienten umspannen, je nach zugrunde liegenden Risikofaktoren, ein weites Feld. In etwa die Hälfte aller Patienten präsentiert sich mit pulmonalen (oder extrapulmonalen) infektiologischen Problemen, wobei ein beträchtlicher Anteil dieser Infektionen auf opportunistische Erreger, wie z. B. invasive Pilzinfektionen bzw. *Pneumocystis jirovecii* (PjP), zurückzuführen ist. Ebenfalls bis zu 50 % aller Patienten weisen als alleinige Auslöser oder als zusätzliche Komplikationen nichtinfektiöse Probleme auf, z. B. maligne Infiltrate durch solide oder (vor allem) hämatologische Malignome, eine therapieassoziierte pulmonale Toxizität durch Chemo‑, Immun-, oder Strahlentherapie, kardiale Kompromittierung, oder andere Ursachen, wie z. B. Tumorlysesyndrom und Hyperleukozytose.

Bis zu 50 % aller Patienten weisen nichtinfektiöse Probleme als Auslöser oder als Komplikation auf

Die adäquate Therapie der jeweiligen Ursache, die oftmals eine enge interdisziplinäre Zusammenarbeit erfordert, determiniert zu einem hohen Ausmaß den Therapieerfolg, sodass die intensivmedizinische Supportivtherapie allein die pulmonale Situation im besten Fall zwar vorübergehend stabilisieren, aber nicht primär verbessern kann.

#### Einfluss einer nichtidentifizierten Ätiologie

Es ist daher nicht überraschend, dass eine nichtidentifizierte Ätiologie des ARV einen starken negativ-prognostischen Einfluss hat. Etliche rezente Daten konnten dies eindrucksvoll belegen. Zwei Landmark-Studien an über 2200 Patienten, eine davon prospektiv durchgeführt, zeigten sogar, dass von allen untersuchten Faktoren eine nichtidentifizierte Ätiologie den *einzigen* potenziell modifizierbaren Risikofaktor für ein schlechteres Outcome darstellt [17, 18]. Während nichtinvasive Oxygenierungsstrategien im Gegensatz zu früheren Ansichten also keinen Einfluss auf den Verlauf der Patienten haben dürften, sollte das Augenmerk heutzutage vielmehr auf eine rigorose Abklärung der ARV-auslösenden Ursachen gelegt werden.

### Erweiterte diagnostische Maßnahmen

Eine randomisierte Studie zum Einsatz einer bronchoalveoläre Lavage (BAL) bei nichtintubierten immunsupprimierten Patienten zeigte, dass ihre Durchführung bei respiratorischer Beeinträchtigung, jedoch bei einer peripheren Sauerstoffsättigung (S_p_O_2_) >90 %, hinsichtlich Intubationsraten und weiterem Verlauf in erfahrenen Händen als sicher anzusehen ist [19]. Jedoch konnte die Ausbeute hinsichtlich einer Ursachenidentifizierung im Vergleich zu einem arbeitsintensiven nichtinvasiven Diagnosealgorithmus *nicht* erhöht werden. In beiden Studiengruppen (nichtinvasive Diagnostik mit oder ohne BAL) lag die Trefferrate bei hohen 80 %. Lediglich Fälle mit PjP konnten unter den gegebenen Bedingungen in den teilnehmenden Studienzentren durch den Einsatz der BAL *schneller* diagnostiziert werden. Daraus könnte gefolgert werden, dass eine BAL in Abwesenheit von Risikofaktoren für eine PjP bei nichtintubierten Krebspatienten mit ARV *nicht* erforderlich ist. Zu den PjP-Risikofaktoren zählen u. a.:

- akute lymphoblastische Leukämie unter Therapie,
- allogene Stammzelltransplantation unter Immunsuppression,
- Steroide >20 mg/kgKG Prednisolonäquivalente für mehr als 4 Wochen,
- fehlende PjP-Prophylaxe bei bestehendem Risiko .

Der in der Studie angewandte „nichtinvasive“ Diagnosealgorithmus wurde in modifizierter Form in den bereits erwähnten Konsensus zur Versorgung kritisch kranker Krebspatienten übernommen (Tab. 2).

**Table 2:** 

Untersuchung	Fragestellung
Blutkulturen	Bakterien/Pilze
Multislice‑/HR-CT	Radiomorphologische Hinweise für spezifische Ätiologie
Echokardiographie	Kardiale Ursache für ARV
Sputum	Bakterien
	Pilze
	Mykobakterien
Induziertes Sputum	*Pneumocystis jirovecii*
Nasopharyngeale Aspirate	RSV, Influenza
Blut-PCR auf Viren	Herpes
	Zytomegalie
	Ebstein-Barr-Virus
Zirkulierendes Galactomannan	Aspergillus
Serologische Tests	*Chlamydia pneumoniae*
	*Mycoplasma pneumoniae*
	*Legionella pneumophila*
Urinantigene	*Legionella pneumophila*
	*Streptococcus pneumoniae*
BAL – Standard	Zytologie und Gram-Färbung
	Bakteriologie inklusive Medien für Legionella spp., Mykobakterien und Pilze
	Calcofluor-Färbung (oder äquivalent) für Pilznachweise
	Immunfluoreszenz für *Pneumocystis jirovecii*
	Galactomannan
	PCR auf Mykobakterien (*Mycobacterium tuberculosis* und atypische Mykobakterien)
BAL – optional	PCR auf Zytomegalie, RSV, Influenza A/B, Parainfluenza, humaner Metapneumovirus, Adenoviren, Varicella-Zoster-Virus und *Pneumocystis jirovecii* (quantitativ)
	Panfugale Pilz-PCR

*ARV* akutes respiratorisches Versagen; *HR-CT* High-resolution-Computertomographie, in der Regel *ohne* Kontrastmittel ausreichend; *RSV* Respiratory Syncytial Virus; *BAL* bronchoalveoläre Lavage; *PCR* Polymerase-Kettenreaktion

Eine differenzierte Herangehensweise, die die Wahl einer ersten Therapie im empirischen Setting, also noch vor Einlangen von definitiven Befunden ermöglicht, wird durch den evaluierten DIRECT-Approach vermittelt. Durch die Informationen

**Figure 1:**
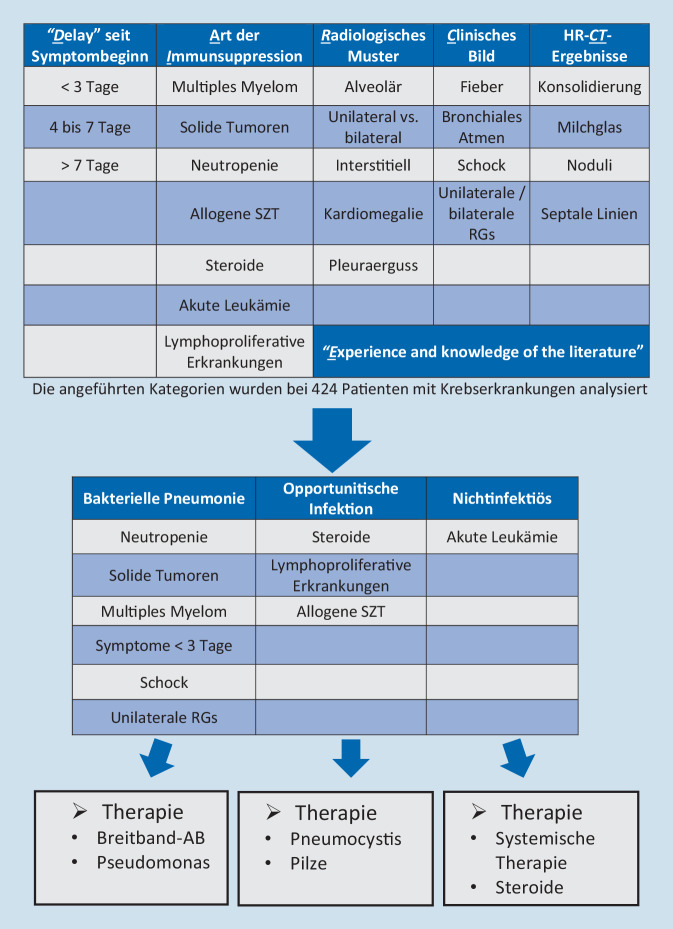

- „**D**elay seit Symptombeginn“,
- „Art der **I**mmunsuppression“,
- „**R**adiologisches Muster“,
- „**E**xperience and knowledge of the literature“,
- „**C**lincial presentation“ sowie
- „Ergebnis der High-resolution-Computertomographie (HR-**CT**)“

kann eine Vortestwahrscheinlichkeit für das Vorliegen der verschiedenen ARV-Ursachen abgeschätzt werden (Abb. 1a, b; [20]). Vor allem der differenzierten Befundung von CT-Bildern kommt vor dem Hintergrund der Anamnese in diesem Zusammenhang eine gewichtige Rolle zu, was die Notwendigkeit einer engen Zusammenarbeit mit den Kollegen der Radiologie betont [21]. Dabei kann nicht nur die Wahrscheinlichkeit für verschiedene, therapierelevante Infektionserregerklassen abgeschätzt werden, sondern auch ein pulmonaler Befall von soliden oder hämatologischen Malignomen. Letzterer erfordert vor einem Therapiebeginn in der Regel jedoch immer eine direkte Diagnostik, es sein denn, es handelt sich um die pulmonale Manifestation einer bereits nachgewiesenen systemischen Erkrankung, wie z. B. ein Hyperleukozytosesyndrom bei akuter Leukämie mit ohnehin bestehender Behandlungsindikation [22].

Die invasive Abklärung von Läsionen durch Biopsien könnte bei unklar verbleibenden Fällen eine sinnvolle Erweiterung der vorhandenen Diagnostik darstellen. Die noch spärlich vorhandenen Daten zeigen eine verhältnismäßig hohe diagnostische Ausbeute mit entsprechender Auswirkungen auf die sich auftuenden Therapieoptionen bei relevanten, jedoch im Verhältnis akzeptabel erscheinenden Komplikationsraten. Vor allem bei blutungsgefährdeten Patienten könnte die Kryobiopsie eine zukünftige Option darstellen [18, 21].

Ein rezenter Review des Nine-i-Netzwerks liefert weitere Ausführungen zum differenzierten diagnostischen Vorgehen bei schweren respiratorischen Infekten des immunsupprimierten Patienten [29].

## Intensivmedizinisch relevante Toxizitäten nach Immuntherapie

In den vergangenen Jahren wurde eine Flut an neuen Immuntherapeutika zur Behandlung von Krebserkrankungen zugelassen. Im Zuge der äußerst vielgestaltigen Toxizität benötigt jeder Dritte mit diesen Medikamenten behandelte Patient eine notfallmedizinische Versorgung. Auch für Intensivmediziner rückt bei Auftreten von Organdysfunktionen die Differenzialdiagnose einer immuntherapieassoziierten Problematik somit zunehmend in den Mittelpunkt. Wiewohl eine ausführliche Besprechung dieses Themas den Fokus dieses Reviews sprengen würde, soll an dieser Stelle in Kürze auf die wichtigsten Punkte eingegangen und auf die entsprechenden Nachschlagwerke bzw. Leitlinien verwiesen werden.

Checkpointinhibitoren werden bei verschiedenen hämatoonkologischen Indikationen angewendet

Insbesondere die sog. Checkpointinhibitoren sind mittlerweile in den verschiedensten hämatologischen und onkologischen Indikationen in Verwendung [23, 24]. Die mit ihnen assoziierte Toxizität betrifft in vielgestaltiger Form den dermatologischen und gastrointestinalen Bereich (Diarrhö, Kolitis, Hepatitis), manifestiert sich in diversen Endokrinopathien, rheumatologischen Krankheitsbildern sowie in der für den Intensivmediziner besonders relevanten Pneumonitis mit möglichem ARV. Seltener befallene Organsysteme stellen das zentrale Nervensystem, die Niere, das Auge, das kardiovaskuläre sowie das blutbildende System dar. Sämtliche unklare Befundkonstellationen sollten zur Abklärung bzw. Kontaktaufnahme mit einem Experten für diese Therapien führen. Die Therapie besteht im einfachsten Fall in einem Absetzen der Medikation, mitunter muss jedoch auch eine (eventuell hochdosierte) Steroidtherapie und bei deren Versagen eine weitere immunsuppressive oder immunmodulatorische Therapie eingeleitet werden. Wiewohl die Evidenz zu diesem Vorgehen in vielen assoziierten Bereichen bis dato noch recht oberflächlich erscheint, sprechen etliche rezente Leitlinien und Übersichtsartikel Empfehlungen zum Management aus [23, 24].

Die sog. Chimeric-antigen-receptor(CAR)-T-Zell-Therapie stellt für erwachsene Patienten mit ungünstig verlaufendem diffus großzelligem B‑Zell-Lymphom sowie für Kinder und junge Erwachsene mit akuter lymphoblastischer B‑Zell-Leukämie eine neue Therapieoption dar. Dabei werden T‑Zellen des Patienten entnommen und im Labor dahingehend verändert, dass sie Antikörper gegen malignomzellassoziierte Antigene exprimieren. Diese werden dann dem Patienten retransfundiert. Während die modifizierten T‑Zellen einerseits in weiterer Folge Krebszellen, die das jeweilige Antigen tragen, attackieren und im besten Fall eliminieren, kommt es in bis zu 90 % der Behandelten zur Ausprägung eines mehr oder minder ausgeprägten „cytokine release syndrome“ (CRS). Dieses führt durch Freisetzung von Zyto- und Chemokinen zu einer starken Aktivierung des Immunsystems, was zu sepsisähnlichen Schockzuständen, ARV und schließlich Multiorganversagen führen kann. Die Therapie besteht in erster Linie aus der intravenösen Verabreichung des humanisierten monoklonalen Antikörpers Tocilizumab, einem Interleukin-6-Rezeptor-Blocker, in zweiter Linie aus hochdosierten Steroidgaben. In bis zu zwei Dritteln der behandelten Patienten kommt es zum Auftreten des immuneffektorzellassoziierten Neurotoxizitätssyndroms (ICANS). Es zeichnet sich durch vielgestaltige klinische Präsentationen von milden Wesensveränderungen, psychiatrischen Komplikationen, fokalen Defiziten bis hin zu Krampfgeschehen, Koma und vital gefährdendem Anstieg des intrazerebralen Drucks aus. Zur Therapie kommen hochdosierte Steroide zum Einsatz [25–28].

Die CAR-T-Zell-Therapie wird bei diffus großzelligen B‑Zell-Lymphomen eingesetzt

Die CAR-T-Zell-Therapie wird bereits in Form zweier von der Europäischen Arzneimittelbehörde (EMA) im Jahr 2018 zugelassener Produkte in der Routinebehandlung von Patienten mit den zuvor erwähnten B‑Zell-Neoplasien eingesetzt, wobei die Verabreichung derzeit noch in überschaubarer Anzahl an Schwerpunktzentren stattfindet [25, 28]. Jedoch läuft eine große Zahl an klinischen Prüfungen bei diversen, auch onkologischen Indikationen mit soliden Tumoren. Sollten in Zukunft entsprechende Präparate für weitere Indikationen zugelassen werden, wird dies das Gesundheitssystem in finanzieller, infrastruktureller und letztlich auch intensivmedizinischer Hinsicht vor relevante Herausforderungen stellen.

## Fazit für die Praxis

- Die Behandlung intensivpflichtiger onkologischer Krankheitsbilder erfordert eine enge Kooperation mit den behandelnden Krebsspezialisten sowie solide Grundkenntnisse der agierenden Intensivmediziner.
- Im Rahmen der Therapiezielentscheidung ist zwischen einem „Full-code“-Management, einem zeitlimitierten ICU Trial sowie einer Ablehnung der intensivmedizinischen Versorgung zu entscheiden.
- Nichtinvasive Beatmungsstrategien beim hypoxischen akuten respiratorischen Versagen (ARV) sind im Vergleich zur konventionellen Sauerstofftherapie nicht mehr klar zu bevorzugen. Ein wesentlicher Fokus sollte bei Auftreten eines ARV auf der rigorosen und differenzierten Abklärung der Ursachen unter Miteinbeziehung nichtinvasiver Diagnosealgorithmen liegen.
- Die Anzahl immuntherapieassoziierter Toxizitäten nimmt stetig zu und ihr Erkennen sowie ihre Therapie sind komplex.
- Der erste deutschsprachige Konsensus zur Versorgung kritisch kranker Krebspatienten bietet bei vielen Fragestellungen Hilfe.
